# Supplementary material for: Mutations in PCYT2 disrupt etherlipid biosynthesis and cause a complex hereditary spastic paraplegia
Source: Brain. 2019 Oct 22;142(11):3382–97. doi: 10.1093/brain/awz291 (PMC6821184; doi:10.1093/brain/awz291)
Supplement: awz291_Supplementary_Data [file awz291_supplementary_data.zip › awz291-Suppl_data/Supplementary_Data4.zip!/Supplementary information 4 - plasma lipidomics.zip!/Plasma lipidomics/Control vs Patient3a/Statistical plots and data/TG[O] species/Volcano plot_alternative.pdf]

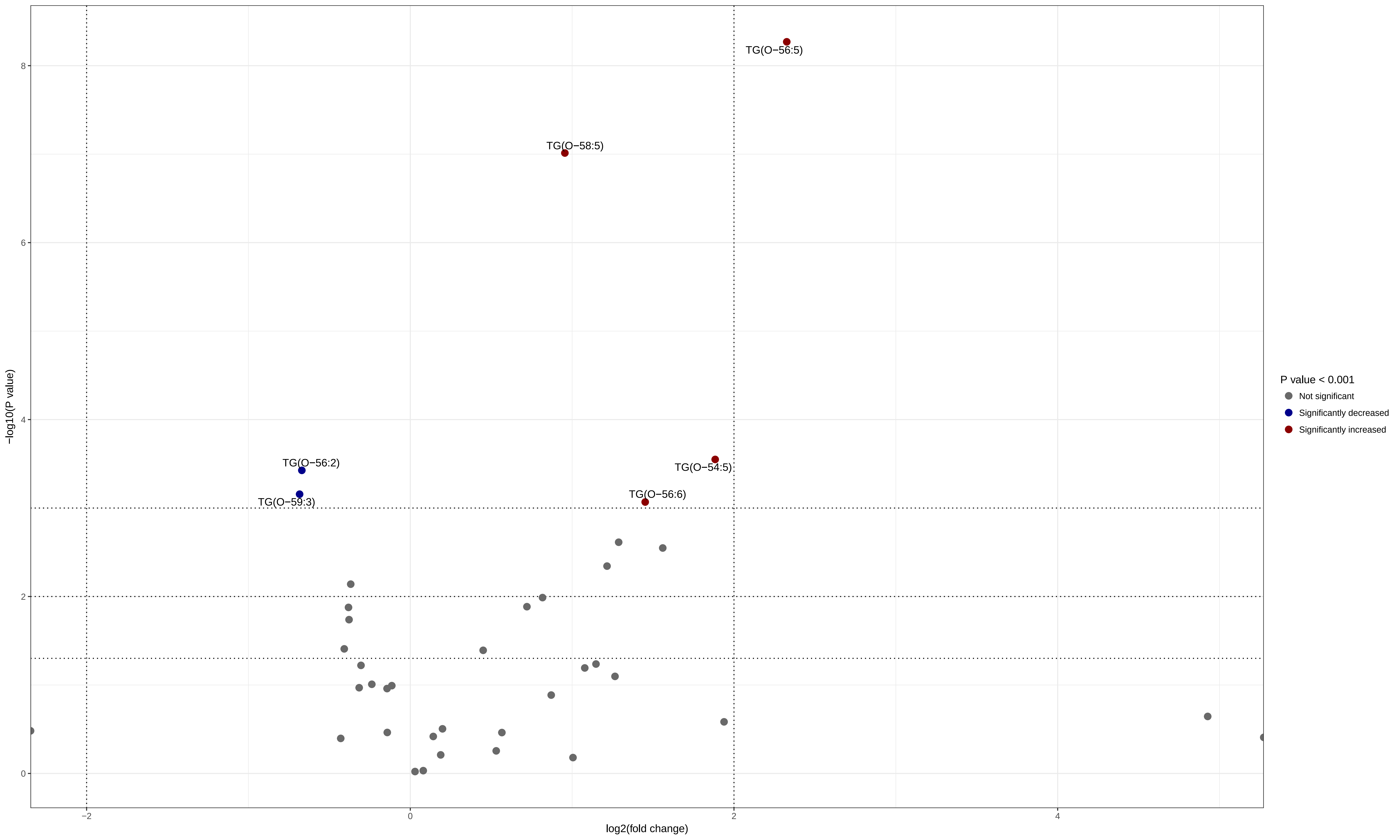

TG(O−56:5)
TG(O−58:5)
TG(O−56:2)
TG(O−59:3)
TG(O−54:5)
TG(O−56:6)
−log10(P value)
log2(fold change)
P value < 0.001
Not significant
Significantly decreased
Significantly increased
